# 2-[5-(4-Fluoro­phen­yl)-3-(4-methyl­phen­yl)-4,5-dihydro-1*H*-pyrazol-1-yl]-4-phenyl-1,3-thia­zole

**DOI:** 10.1107/S1600536813004339

**Published:** 2013-02-16

**Authors:** Bakr F. Abdel-Wahab, Hanan A. Mohamed, Seik Weng Ng, Edward R. T. Tiekink

**Affiliations:** a Applied Organic Chemistry Department, National Research Centre, Dokki, 12622 Giza, Egypt; b Department of Chemistry, University of Malaya, 50603 Kuala Lumpur, Malaysia; c Chemistry Department, Faculty of Science, King Abdulaziz University, PO Box 80203 Jeddah, Saudi Arabia

## Abstract

In the title compound, C_25_H_20_FN_3_S, two independent mol­ecules comprise the asymmetric unit, which differ in the relative orientation of the fluoro­benzene ring with respect to the pyrazole ring to which it is attached [dihedral angles = 89.39 (17) and 75.23 (16)° in the two mol­ecules]. Each pyrazole ring adopts an envelope conformation with the methine C atom being the flap atom. There are additional twists in the mol­ecules, *e.g*. between the five-membered rings [dihedral angles = 18.23 (16) and 17.84 (16)°] and between the thia­zole and attached phenyl ring [10.26 (16) and 20.87 (15)°]. Overall, each mol­ecule has a T-shape. In the crystal, mol­ecules are connected into a three-dimensional architecture by weak C—H⋯π inter­actions.

## Related literature

For the biological activity of pyrazolin-1-yl­thia­zoles, see: Abdel-Wahab *et al.* (2009 ▶, 2012 ▶); Chimenti *et al.* (2010 ▶). For a related structure, see: Fun *et al.* (2011 ▶).
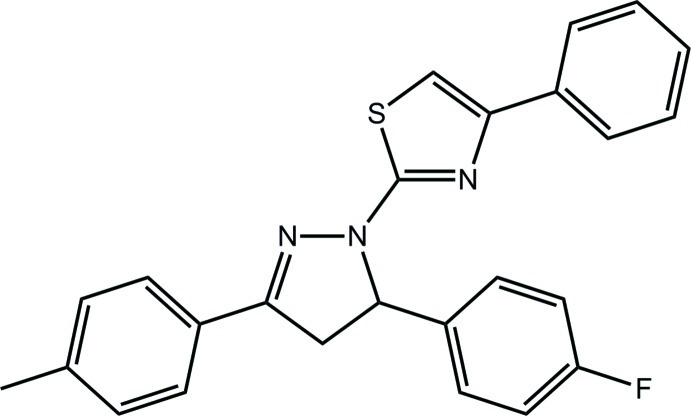


## Experimental

### 

#### Crystal data

C_25_H_20_FN_3_S
*M* *_r_* = 413.50
Monoclinic, 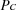

*a* = 5.7563 (3) Å
*b* = 24.1214 (12) Å
*c* = 15.1827 (8) Å
β = 90.948 (5)°
*V* = 2107.83 (19) Å^3^
*Z* = 4
Mo *K*α radiation
μ = 0.18 mm^−1^
*T* = 295 K
0.40 × 0.30 × 0.20 mm

#### Data collection

Agilent SuperNova Dual diffractometer with an Atlas detector
Absorption correction: multi-scan (*CrysAlis PRO*; Agilent, 2011 ▶) *T*_min_ = 0.837, *T*_max_ = 1.000
22651 measured reflections
9054 independent reflections
5841 reflections with *I* > 2σ(*I*)
*R*_int_ = 0.042

#### Refinement

*R*[*F* ^2^ > 2σ(*F* ^2^)] = 0.048
*wR*(*F* ^2^) = 0.117
*S* = 0.99
9054 reflections
543 parameters
2 restraints
H-atom parameters constrained
Δρ_max_ = 0.14 e Å^−3^
Δρ_min_ = −0.16 e Å^−3^
Absolute structure: Flack (1983 ▶), 4165 Friedel pairs
Flack parameter: −0.06 (6)

### 

Data collection: *CrysAlis PRO* (Agilent, 2011 ▶); cell refinement: *CrysAlis PRO*; data reduction: *CrysAlis PRO*; program(s) used to solve structure: *SHELXS97* (Sheldrick, 2008 ▶); program(s) used to refine structure: *SHELXL97* (Sheldrick, 2008 ▶); molecular graphics: *ORTEP-3 for Windows* (Farrugia, 2012 ▶), *QMol* (Gans & Shalloway, 2001 ▶) and *DIAMOND* (Brandenburg, 2006 ▶); software used to prepare material for publication: *publCIF* (Westrip, 2010 ▶).

## Supplementary Material

Crystal structure: contains datablock(s) global, I. DOI: 10.1107/S1600536813004339/hg5292sup1.cif

Structure factors: contains datablock(s) I. DOI: 10.1107/S1600536813004339/hg5292Isup2.hkl

Supplementary material file. DOI: 10.1107/S1600536813004339/hg5292Isup3.cml

Additional supplementary materials:  crystallographic information; 3D view; checkCIF report

## Figures and Tables

**Table 1:** Hydrogen-bond geometry (Å, °) *Cg*1, *Cg*2, *Cg*3 and *Cg*4 are the centroids of the C26–C31, C45–C50, S2,N4,C32–C34 and C1–C6 rings, respectively.

*D*—H⋯*A*	*D*—H	H⋯*A*	*D*⋯*A*	*D*—H⋯*A*
C15—H15⋯*Cg*1	0.93	2.75	3.537 (3)	143
C19—H19*A*⋯*Cg*2^i^	0.96	2.94	3.596 (4)	127
C35—H35⋯*Cg*3^ii^	0.98	2.94	3.843 (3)	153
C42—H42⋯*Cg*4^iii^	0.93	2.86	3.567 (3)	134

Symmetry codes: (i) 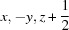; (ii) 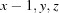; (iii) 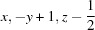.

**Fig. 1.**
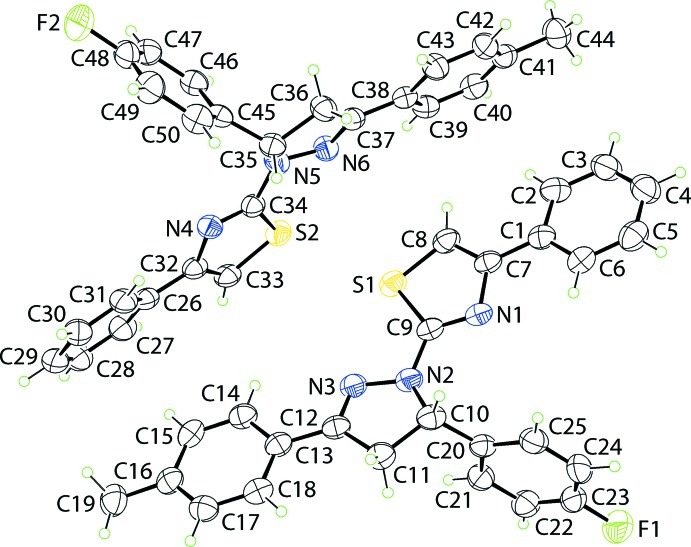
The molecular structure of (I) showing the atom-labelling scheme and displacement ellipsoids at the 35% probability level.

**Fig. 2.**
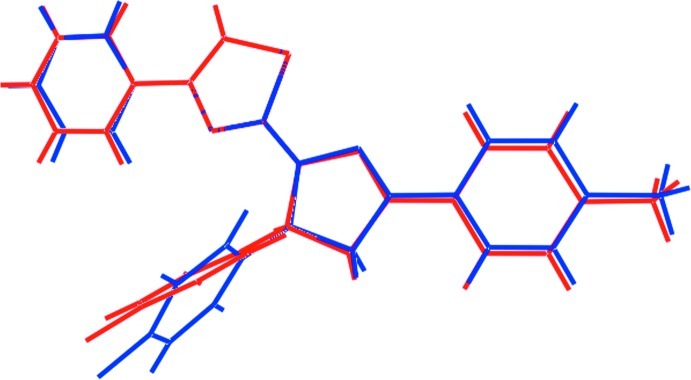
Overlay diagram of the two independent molecules in (I) with the S1-containing molecule illustrated in red. The molecules are overlaid so that the thiazolyl rings are superimposed.

**Fig. 3.**
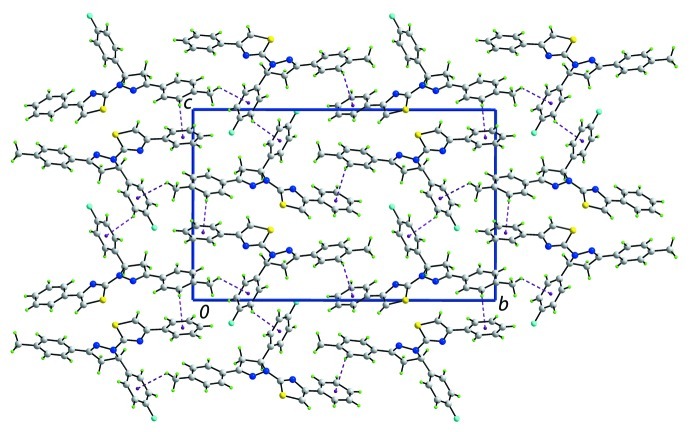
A view of the crystal packing in projection down the a axis. The C—H···π interactions are shown as purple dashed lines.

## supplementary crystallographic information

### Comment

The title compound (I) was investigated in relation to the established biological activities exhibited by pyrazolin-1-ylthiazoles (Abdel-Wahab *et al.*, 2012; Abdel-Wahab *et al.*, 2009; Chimenti *et al.*, 2010).

Two independent molecules comprise the crystallographic asymmetric unit of (I), Fig. 1. As judged from the overlay diagram, Fig. 2, the central residues are super-imposable with the major difference relating to the relative orientations of the fluorobenzene rings with the dihedral angle formed with the least-squares plane through the pyrazolyl ring being 89.39 (17)° for the S1-containing molecule and 75.23 (16)° for the second molecule. Each pyrazolyl ring adopts an envelope conformation with the methine-C atom, *i.e*. C10 and C35, being the flap atom in each case. With the exception of the near perpendicular relationship between the pyrazolyl and fluorobenzene rings, the remaining components of the molecule exhibit relatively small twists as indicated by the dihedral angles formed between the five-membered rings [18.23 (16); 17.84 (16)°], the thiazolyl and attached phenyl ring [10.26 (16); 20.87 (15)°], and between the pyrazolyl and appended tolyl ring [2.37 (16); 1.96 (16)°]. Overall, the molecule has the shape of the letter T and resembles the structure of a literature precedent (Fun *et al.*, 2011).

The crystal packing is dominated by C—H···π interactions that lead to a three-dimensional architecture, Fig. 3 and Table 1.

### Experimental

A mixture of 5-(4-fluorophenyl)-3-*p*-tolyl-4,5-dihydro-1*H*-pyrazole-1-carbothioamide (0.31 g, 0.001 *M*) and 2-bromo-1-phenylethanone (0.2 g, 0.001 *M*) in anhydrous ethanol (30 ml) was heated under reflux for about 4 h. The resultant solid was filtered and dried. Re-crystallization was by slow evaporation of a DMF solution of (I) which yielded colourless crystals in 59% yield. *M*.pt. 418–420 K.

### Refinement

Carbon-bound H-atoms were placed in calculated positions (C—H = 0.93 to 0.98 Å) and were included in the refinement in the riding model approximation, with *U*_iso_(H) = 1.2–1.5*U*_equiv_(C).

### Crystal data

**Table:**

C_25_H_20_FN_3_S	*F*(000) = 864
*M**_r_* = 413.50	*D*_x_ = 1.303 Mg m^−^^3^
Monoclinic, *P**c*	Mo *K*α radiation, λ = 0.71073 Å
Hall symbol: P -2yc	Cell parameters from 5001 reflections
*a* = 5.7563 (3) Å	θ = 2.9–27.5°
*b* = 24.1214 (12) Å	µ = 0.18 mm^−^^1^
*c* = 15.1827 (8) Å	*T* = 295 K
β = 90.948 (5)°	Prism, yellow
*V* = 2107.83 (19) Å^3^	0.40 × 0.30 × 0.20 mm
*Z* = 4	

### Data collection

**Table:**

Agilent SuperNova Dual diffractometer with an Atlas detector	9054 independent reflections
Radiation source: SuperNova (Mo) X-ray Source	5841 reflections with *I* > 2σ(*I*)
Mirror monochromator	*R*_int_ = 0.042
Detector resolution: 10.4041 pixels mm^-1^	θ_max_ = 27.6°, θ_min_ = 2.9°
ω scan	*h* = −7→7
Absorption correction: multi-scan (*CrysAlis PRO*; Agilent, 2011)	*k* = −31→31
*T*_min_ = 0.837, *T*_max_ = 1.000	*l* = −19→19
22651 measured reflections	

### Refinement

**Table:**

Refinement on *F*^2^	Secondary atom site location: difference Fourier map
Least-squares matrix: full	Hydrogen site location: inferred from neighbouring sites
*R*[*F*^2^ > 2σ(*F*^2^)] = 0.048	H-atom parameters constrained
*wR*(*F*^2^) = 0.117	*w* = 1/[σ^2^(*F*_o_^2^) + (0.0492*P*)^2^] where *P* = (*F*_o_^2^ + 2*F*_c_^2^)/3
*S* = 0.99	(Δ/σ)_max_ < 0.001
9054 reflections	Δρ_max_ = 0.14 e Å^−^^3^
543 parameters	Δρ_min_ = −0.16 e Å^−^^3^
2 restraints	Absolute structure: Flack (1983), 4165 Friedel pairs
Primary atom site location: structure-invariant direct methods	Flack parameter: −0.06 (6)

### Special details

**Table:**

Geometry. All e.s.d.'s (except the e.s.d. in the dihedral angle between two l.s. planes) are estimated using the full covariance matrix. The cell e.s.d.'s are taken into account individually in the estimation of e.s.d.'s in distances, angles and torsion angles; correlations between e.s.d.'s in cell parameters are only used when they are defined by crystal symmetry. An approximate (isotropic) treatment of cell e.s.d.'s is used for estimating e.s.d.'s involving l.s. planes.
Refinement. Refinement of *F*^2^ against ALL reflections. The weighted *R*-factor *wR* and goodness of fit *S* are based on *F*^2^, conventional *R*-factors *R* are based on *F*, with *F* set to zero for negative *F*^2^. The threshold expression of *F*^2^ > σ(*F*^2^) is used only for calculating *R*-factors(gt) *etc*. and is not relevant to the choice of reflections for refinement. *R*-factors based on *F*^2^ are statistically about twice as large as those based on *F*, and *R*- factors based on ALL data will be even larger.

### Fractional atomic coordinates and isotropic or equivalent isotropic displacement parameters (Å^2^)

**Table:**

	*x*	*y*	*z*	*U*_iso_*/*U*_eq_	
S1	0.49969 (14)	0.29737 (3)	0.50008 (6)	0.0662 (2)	
S2	1.01138 (13)	0.25628 (3)	0.36822 (6)	0.0666 (2)	
F1	−0.0507 (5)	0.34215 (9)	0.99078 (17)	0.1149 (8)	
F2	0.5094 (5)	0.12749 (9)	−0.12101 (16)	0.1042 (7)	
N1	0.1354 (4)	0.32997 (10)	0.58332 (16)	0.0579 (6)	
N2	0.2741 (4)	0.24238 (11)	0.62782 (17)	0.0638 (7)	
N3	0.4085 (4)	0.19780 (11)	0.60017 (17)	0.0621 (7)	
N4	0.8192 (4)	0.17483 (10)	0.28401 (16)	0.0585 (6)	
N5	0.6904 (4)	0.26272 (10)	0.23727 (18)	0.0635 (7)	
N6	0.6916 (4)	0.31858 (10)	0.26139 (16)	0.0590 (7)	
C1	0.0526 (5)	0.42283 (12)	0.52259 (19)	0.0571 (8)	
C2	0.1262 (6)	0.46954 (15)	0.4761 (2)	0.0740 (9)	
H2A	0.2631	0.4680	0.4445	0.089*	
C3	−0.0020 (7)	0.51781 (16)	0.4767 (3)	0.0822 (11)	
H3	0.0519	0.5489	0.4470	0.099*	
C4	−0.2069 (7)	0.52053 (16)	0.5204 (3)	0.0835 (11)	
H4	−0.2947	0.5529	0.5192	0.100*	
C5	−0.2824 (6)	0.47491 (16)	0.5663 (2)	0.0758 (10)	
H5	−0.4214	0.4766	0.5965	0.091*	
C6	−0.1532 (6)	0.42654 (14)	0.5678 (2)	0.0653 (8)	
H6	−0.2055	0.3962	0.5995	0.078*	
C7	0.1944 (5)	0.37188 (12)	0.52465 (19)	0.0554 (7)	
C8	0.3820 (6)	0.36115 (13)	0.4756 (2)	0.0643 (8)	
H8	0.4406	0.3853	0.4336	0.077*	
C9	0.2832 (5)	0.28972 (13)	0.57623 (19)	0.0559 (7)	
C10	0.0630 (5)	0.22505 (13)	0.6747 (2)	0.0606 (8)	
H10	−0.0735	0.2297	0.6360	0.073*	
C11	0.1131 (6)	0.16282 (13)	0.6878 (2)	0.0664 (9)	
H11A	0.1453	0.1544	0.7493	0.080*	
H11B	−0.0168	0.1404	0.6673	0.080*	
C12	0.3231 (5)	0.15310 (13)	0.6329 (2)	0.0569 (7)	
C13	0.4368 (5)	0.09940 (13)	0.61754 (19)	0.0566 (7)	
C14	0.6347 (5)	0.09546 (14)	0.5673 (2)	0.0640 (8)	
H14	0.6926	0.1270	0.5402	0.077*	
C15	0.7465 (6)	0.04580 (14)	0.5568 (2)	0.0671 (9)	
H15	0.8800	0.0445	0.5232	0.081*	
C16	0.6653 (5)	−0.00304 (13)	0.5954 (2)	0.0618 (8)	
C17	0.4650 (6)	0.00095 (14)	0.6425 (2)	0.0659 (8)	
H17	0.4040	−0.0309	0.6677	0.079*	
C18	0.3500 (6)	0.05102 (14)	0.6539 (2)	0.0652 (8)	
H18	0.2140	0.0521	0.6861	0.078*	
C19	0.7985 (6)	−0.05603 (14)	0.5865 (3)	0.0827 (11)	
H19A	0.7180	−0.0853	0.6161	0.124*	
H19B	0.9506	−0.0516	0.6125	0.124*	
H19C	0.8123	−0.0651	0.5253	0.124*	
C20	0.0305 (5)	0.25749 (12)	0.7580 (2)	0.0542 (7)	
C21	0.1996 (5)	0.25682 (13)	0.8234 (2)	0.0637 (8)	
H21	0.3345	0.2365	0.8146	0.076*	
C22	0.1749 (7)	0.28550 (14)	0.9016 (2)	0.0733 (9)	
H22	0.2902	0.2844	0.9452	0.088*	
C23	−0.0195 (7)	0.31483 (13)	0.9126 (2)	0.0731 (10)	
C24	−0.1901 (7)	0.31738 (15)	0.8503 (3)	0.0816 (11)	
H24	−0.3236	0.3380	0.8603	0.098*	
C25	−0.1649 (6)	0.28920 (14)	0.7719 (3)	0.0709 (9)	
H25	−0.2800	0.2916	0.7284	0.085*	
C26	1.0106 (5)	0.09110 (13)	0.34701 (18)	0.0561 (7)	
C27	1.2113 (6)	0.06737 (16)	0.3842 (2)	0.0696 (9)	
H27	1.3308	0.0901	0.4048	0.084*	
C28	1.2335 (6)	0.01085 (17)	0.3907 (2)	0.0775 (10)	
H28	1.3676	−0.0043	0.4157	0.093*	
C29	1.0595 (6)	−0.02337 (15)	0.3605 (2)	0.0737 (10)	
H29	1.0758	−0.0617	0.3645	0.088*	
C30	0.8601 (6)	−0.00060 (15)	0.3242 (2)	0.0731 (9)	
H30	0.7405	−0.0236	0.3045	0.088*	
C31	0.8373 (6)	0.05605 (13)	0.3171 (2)	0.0643 (8)	
H31	0.7029	0.0709	0.2917	0.077*	
C32	0.9822 (5)	0.15215 (13)	0.34277 (19)	0.0551 (7)	
C33	1.0981 (6)	0.18976 (14)	0.3925 (2)	0.0644 (8)	
H33	1.2110	0.1808	0.4347	0.077*	
C34	0.8228 (5)	0.22829 (13)	0.2909 (2)	0.0550 (7)	
C35	0.4651 (5)	0.24534 (12)	0.1964 (2)	0.0559 (8)	
H35	0.3740	0.2246	0.2392	0.067*	
C36	0.3531 (5)	0.30230 (12)	0.1797 (2)	0.0619 (8)	
H36A	0.3483	0.3109	0.1173	0.074*	
H36B	0.1964	0.3034	0.2021	0.074*	
C37	0.5083 (5)	0.34182 (12)	0.22889 (19)	0.0544 (7)	
C38	0.4623 (5)	0.40098 (12)	0.23963 (19)	0.0531 (7)	
C39	0.6205 (5)	0.43590 (13)	0.2823 (2)	0.0608 (8)	
H39	0.7594	0.4216	0.3046	0.073*	
C40	0.5724 (6)	0.49193 (13)	0.2918 (2)	0.0673 (9)	
H40	0.6797	0.5146	0.3207	0.081*	
C41	0.3681 (6)	0.51480 (13)	0.2591 (2)	0.0619 (8)	
C42	0.2136 (6)	0.48056 (13)	0.2167 (2)	0.0646 (8)	
H42	0.0760	0.4952	0.1938	0.077*	
C43	0.2585 (5)	0.42450 (12)	0.2075 (2)	0.0629 (8)	
H43	0.1493	0.4021	0.1790	0.075*	
C44	0.3189 (8)	0.57637 (13)	0.2676 (3)	0.0901 (12)	
H44A	0.3506	0.5880	0.3270	0.135*	
H44B	0.1588	0.5834	0.2529	0.135*	
H44C	0.4163	0.5966	0.2281	0.135*	
C45	0.4915 (5)	0.21170 (11)	0.1141 (2)	0.0520 (7)	
C46	0.6698 (5)	0.21963 (12)	0.0566 (2)	0.0607 (8)	
H46	0.7872	0.2447	0.0711	0.073*	
C47	0.6789 (6)	0.19115 (13)	−0.0227 (2)	0.0671 (8)	
H47	0.8014	0.1963	−0.0610	0.081*	
C48	0.5029 (7)	0.15541 (13)	−0.0426 (2)	0.0689 (9)	
C49	0.3243 (7)	0.14560 (14)	0.0122 (3)	0.0758 (10)	
H49	0.2083	0.1204	−0.0031	0.091*	
C50	0.3179 (6)	0.17378 (13)	0.0911 (3)	0.0696 (9)	
H50	0.1964	0.1674	0.1294	0.084*	

### Atomic displacement parameters (Å^2^)

**Table:**

	*U*^11^	*U*^22^	*U*^33^	*U*^12^	*U*^13^	*U*^23^
S1	0.0668 (5)	0.0716 (5)	0.0607 (5)	−0.0069 (4)	0.0122 (4)	−0.0014 (4)
S2	0.0692 (5)	0.0773 (5)	0.0529 (5)	0.0069 (4)	−0.0124 (4)	−0.0081 (4)
F1	0.177 (2)	0.0779 (13)	0.0908 (16)	−0.0016 (15)	0.0292 (17)	−0.0204 (13)
F2	0.1388 (19)	0.0879 (13)	0.0854 (15)	−0.0013 (14)	−0.0106 (15)	−0.0298 (13)
N1	0.0642 (15)	0.0663 (15)	0.0432 (14)	−0.0055 (13)	0.0048 (12)	0.0038 (12)
N2	0.0670 (16)	0.0699 (16)	0.0547 (16)	0.0006 (13)	0.0104 (13)	0.0083 (13)
N3	0.0647 (16)	0.0700 (17)	0.0518 (16)	0.0040 (13)	0.0053 (13)	0.0062 (13)
N4	0.0555 (14)	0.0685 (16)	0.0512 (15)	0.0116 (12)	−0.0105 (12)	0.0020 (13)
N5	0.0650 (15)	0.0591 (15)	0.0655 (17)	0.0098 (12)	−0.0209 (13)	−0.0019 (13)
N6	0.0581 (14)	0.0603 (14)	0.0582 (16)	0.0021 (12)	−0.0104 (13)	−0.0035 (12)
C1	0.0609 (18)	0.0634 (18)	0.0467 (17)	−0.0098 (15)	−0.0093 (14)	−0.0040 (14)
C2	0.072 (2)	0.083 (2)	0.067 (2)	−0.0057 (19)	0.0038 (18)	0.0146 (18)
C3	0.086 (3)	0.077 (2)	0.084 (3)	0.007 (2)	0.001 (2)	0.021 (2)
C4	0.095 (3)	0.075 (2)	0.080 (3)	0.012 (2)	−0.013 (2)	0.003 (2)
C5	0.065 (2)	0.097 (3)	0.065 (2)	−0.001 (2)	0.0001 (17)	−0.010 (2)
C6	0.067 (2)	0.074 (2)	0.0554 (19)	−0.0061 (17)	0.0055 (16)	−0.0052 (16)
C7	0.0610 (18)	0.0633 (18)	0.0418 (16)	−0.0145 (15)	−0.0016 (14)	−0.0017 (14)
C8	0.071 (2)	0.0666 (18)	0.0557 (19)	−0.0123 (16)	0.0138 (17)	0.0013 (16)
C9	0.0605 (17)	0.0673 (18)	0.0400 (16)	−0.0059 (15)	−0.0001 (14)	0.0010 (14)
C10	0.0497 (16)	0.077 (2)	0.0553 (19)	−0.0016 (15)	−0.0042 (15)	0.0081 (16)
C11	0.066 (2)	0.070 (2)	0.063 (2)	−0.0097 (16)	0.0016 (17)	0.0006 (16)
C12	0.0578 (17)	0.0697 (19)	0.0431 (16)	−0.0068 (15)	−0.0077 (14)	0.0023 (15)
C13	0.0549 (17)	0.0694 (19)	0.0454 (17)	−0.0034 (15)	−0.0060 (14)	−0.0019 (15)
C14	0.067 (2)	0.070 (2)	0.0549 (19)	−0.0090 (16)	−0.0011 (16)	0.0005 (15)
C15	0.0645 (19)	0.083 (2)	0.0536 (19)	−0.0008 (18)	0.0027 (16)	−0.0129 (18)
C16	0.066 (2)	0.070 (2)	0.0495 (18)	−0.0079 (16)	−0.0076 (16)	−0.0106 (16)
C17	0.072 (2)	0.0660 (19)	0.060 (2)	−0.0098 (17)	−0.0073 (17)	0.0031 (16)
C18	0.0619 (18)	0.078 (2)	0.055 (2)	−0.0063 (17)	0.0025 (15)	0.0022 (17)
C19	0.093 (3)	0.076 (2)	0.079 (3)	0.002 (2)	−0.003 (2)	−0.018 (2)
C20	0.0501 (16)	0.0601 (16)	0.0524 (18)	−0.0029 (14)	0.0026 (14)	0.0095 (14)
C21	0.0591 (18)	0.0691 (19)	0.063 (2)	0.0061 (15)	−0.0053 (16)	0.0031 (16)
C22	0.086 (2)	0.074 (2)	0.061 (2)	−0.0015 (19)	−0.0048 (19)	−0.0029 (18)
C23	0.101 (3)	0.0535 (18)	0.065 (2)	−0.0041 (19)	0.018 (2)	−0.0044 (17)
C24	0.079 (2)	0.072 (2)	0.094 (3)	0.0243 (19)	0.025 (2)	0.012 (2)
C25	0.064 (2)	0.076 (2)	0.072 (2)	0.0103 (17)	0.0082 (18)	0.0179 (19)
C26	0.0527 (17)	0.0735 (19)	0.0421 (16)	0.0104 (15)	−0.0006 (13)	0.0056 (14)
C27	0.0572 (18)	0.086 (2)	0.065 (2)	0.0125 (16)	−0.0054 (16)	0.0035 (17)
C28	0.072 (2)	0.090 (3)	0.070 (2)	0.032 (2)	−0.0053 (18)	0.011 (2)
C29	0.089 (2)	0.075 (2)	0.057 (2)	0.021 (2)	−0.0019 (19)	0.0079 (18)
C30	0.082 (2)	0.074 (2)	0.063 (2)	0.0094 (18)	−0.0098 (18)	0.0033 (17)
C31	0.0614 (18)	0.075 (2)	0.056 (2)	0.0131 (17)	−0.0110 (15)	0.0077 (16)
C32	0.0491 (16)	0.0733 (19)	0.0427 (16)	0.0079 (14)	−0.0031 (13)	0.0041 (15)
C33	0.0628 (18)	0.084 (2)	0.0461 (18)	0.0082 (16)	−0.0112 (15)	0.0038 (16)
C34	0.0531 (16)	0.0656 (18)	0.0464 (17)	0.0087 (15)	−0.0025 (13)	−0.0003 (15)
C35	0.0519 (16)	0.0605 (17)	0.0549 (18)	−0.0004 (13)	−0.0085 (14)	0.0037 (14)
C36	0.0619 (18)	0.0592 (18)	0.064 (2)	0.0058 (15)	−0.0104 (16)	0.0040 (15)
C37	0.0552 (17)	0.0613 (17)	0.0465 (17)	0.0025 (14)	−0.0024 (14)	0.0037 (14)
C38	0.0543 (16)	0.0603 (17)	0.0447 (16)	−0.0033 (14)	0.0028 (13)	0.0045 (14)
C39	0.0578 (18)	0.0704 (19)	0.0540 (18)	−0.0023 (15)	−0.0047 (15)	0.0073 (16)
C40	0.074 (2)	0.067 (2)	0.061 (2)	−0.0159 (17)	−0.0003 (18)	−0.0023 (17)
C41	0.073 (2)	0.0603 (18)	0.0528 (18)	−0.0029 (16)	0.0070 (16)	0.0015 (15)
C42	0.0629 (19)	0.0662 (19)	0.065 (2)	0.0081 (16)	−0.0029 (16)	0.0008 (17)
C43	0.0617 (18)	0.0603 (18)	0.066 (2)	0.0000 (15)	−0.0092 (16)	−0.0009 (16)
C44	0.116 (3)	0.062 (2)	0.092 (3)	0.003 (2)	−0.004 (2)	−0.004 (2)
C45	0.0508 (16)	0.0504 (15)	0.0544 (18)	−0.0006 (13)	−0.0078 (15)	0.0096 (13)
C46	0.0610 (18)	0.0540 (16)	0.067 (2)	−0.0112 (14)	−0.0064 (17)	0.0056 (16)
C47	0.072 (2)	0.0654 (18)	0.064 (2)	−0.0014 (17)	0.0083 (17)	0.0076 (17)
C48	0.086 (2)	0.0556 (18)	0.065 (2)	0.0055 (17)	−0.012 (2)	−0.0073 (16)
C49	0.078 (2)	0.068 (2)	0.081 (3)	−0.0147 (18)	−0.013 (2)	−0.0119 (19)
C50	0.0591 (18)	0.0695 (19)	0.080 (2)	−0.0128 (16)	−0.0003 (17)	0.0046 (19)

### Geometric parameters (Å, º)

**Table:**

S1—C8	1.719 (3)	C20—C25	1.379 (4)
S1—C9	1.724 (3)	C21—C22	1.384 (5)
S2—C33	1.719 (3)	C21—H21	0.9300
S2—C34	1.723 (3)	C22—C23	1.337 (5)
F1—C23	1.371 (4)	C22—H22	0.9300
F2—C48	1.369 (4)	C23—C24	1.353 (5)
N1—C9	1.297 (4)	C24—C25	1.381 (5)
N1—C7	1.393 (4)	C24—H24	0.9300
N2—C9	1.386 (4)	C25—H25	0.9300
N2—N3	1.393 (4)	C26—C31	1.379 (4)
N2—C10	1.479 (4)	C26—C27	1.400 (4)
N3—C12	1.288 (4)	C26—C32	1.483 (4)
N4—C34	1.294 (4)	C27—C28	1.373 (5)
N4—C32	1.396 (3)	C27—H27	0.9300
N5—C34	1.384 (3)	C28—C29	1.370 (5)
N5—N6	1.396 (3)	C28—H28	0.9300
N5—C35	1.489 (3)	C29—C30	1.379 (5)
N6—C37	1.286 (3)	C29—H29	0.9300
C1—C6	1.381 (4)	C30—C31	1.377 (5)
C1—C2	1.399 (4)	C30—H30	0.9300
C1—C7	1.476 (4)	C31—H31	0.9300
C2—C3	1.379 (5)	C32—C33	1.349 (4)
C2—H2A	0.9300	C33—H33	0.9300
C3—C4	1.364 (5)	C35—C45	1.500 (4)
C3—H3	0.9300	C35—C36	1.537 (4)
C4—C5	1.377 (5)	C35—H35	0.9800
C4—H4	0.9300	C36—C37	1.498 (4)
C5—C6	1.384 (5)	C36—H36A	0.9700
C5—H5	0.9300	C36—H36B	0.9700
C6—H6	0.9300	C37—C38	1.461 (4)
C7—C8	1.347 (4)	C38—C43	1.385 (4)
C8—H8	0.9300	C38—C39	1.392 (4)
C10—C20	1.502 (4)	C39—C40	1.388 (4)
C10—C11	1.541 (4)	C39—H39	0.9300
C10—H10	0.9800	C40—C41	1.384 (4)
C11—C12	1.498 (5)	C40—H40	0.9300
C11—H11A	0.9700	C41—C42	1.366 (4)
C11—H11B	0.9700	C41—C44	1.518 (4)
C12—C13	1.472 (4)	C42—C43	1.384 (4)
C13—C14	1.384 (4)	C42—H42	0.9300
C13—C18	1.388 (4)	C43—H43	0.9300
C14—C15	1.371 (4)	C44—H44A	0.9600
C14—H14	0.9300	C44—H44B	0.9600
C15—C16	1.400 (5)	C44—H44C	0.9600
C15—H15	0.9300	C45—C46	1.372 (4)
C16—C17	1.370 (4)	C45—C50	1.395 (4)
C16—C19	1.498 (5)	C46—C47	1.388 (5)
C17—C18	1.390 (5)	C46—H46	0.9300
C17—H17	0.9300	C47—C48	1.360 (5)
C18—H18	0.9300	C47—H47	0.9300
C19—H19A	0.9600	C48—C49	1.354 (5)
C19—H19B	0.9600	C49—C50	1.378 (5)
C19—H19C	0.9600	C49—H49	0.9300
C20—C21	1.379 (4)	C50—H50	0.9300
			
C8—S1—C9	87.38 (16)	C23—C24—C25	119.7 (3)
C33—S2—C34	87.58 (14)	C23—C24—H24	120.2
C9—N1—C7	108.8 (3)	C25—C24—H24	120.2
C9—N2—N3	116.0 (3)	C20—C25—C24	120.2 (3)
C9—N2—C10	123.0 (2)	C20—C25—H25	119.9
N3—N2—C10	113.1 (2)	C24—C25—H25	119.9
C12—N3—N2	108.2 (3)	C31—C26—C27	118.0 (3)
C34—N4—C32	109.2 (2)	C31—C26—C32	121.1 (2)
C34—N5—N6	115.1 (2)	C27—C26—C32	120.9 (3)
C34—N5—C35	123.0 (2)	C28—C27—C26	120.7 (3)
N6—N5—C35	112.4 (2)	C28—C27—H27	119.7
C37—N6—N5	108.6 (2)	C26—C27—H27	119.7
C6—C1—C2	117.9 (3)	C29—C28—C27	120.5 (3)
C6—C1—C7	121.5 (3)	C29—C28—H28	119.7
C2—C1—C7	120.6 (3)	C27—C28—H28	119.7
C3—C2—C1	120.7 (4)	C28—C29—C30	119.5 (3)
C3—C2—H2A	119.6	C28—C29—H29	120.2
C1—C2—H2A	119.6	C30—C29—H29	120.2
C4—C3—C2	120.7 (4)	C31—C30—C29	120.3 (3)
C4—C3—H3	119.6	C31—C30—H30	119.9
C2—C3—H3	119.6	C29—C30—H30	119.9
C3—C4—C5	119.4 (4)	C30—C31—C26	121.0 (3)
C3—C4—H4	120.3	C30—C31—H31	119.5
C5—C4—H4	120.3	C26—C31—H31	119.5
C4—C5—C6	120.5 (4)	C33—C32—N4	114.5 (3)
C4—C5—H5	119.7	C33—C32—C26	126.2 (2)
C6—C5—H5	119.7	N4—C32—C26	119.3 (3)
C5—C6—C1	120.8 (3)	C32—C33—S2	111.6 (2)
C5—C6—H6	119.6	C32—C33—H33	124.2
C1—C6—H6	119.6	S2—C33—H33	124.2
C8—C7—N1	114.9 (3)	N4—C34—N5	122.8 (3)
C8—C7—C1	126.6 (3)	N4—C34—S2	117.1 (2)
N1—C7—C1	118.5 (3)	N5—C34—S2	120.0 (2)
C7—C8—S1	111.6 (2)	N5—C35—C45	113.6 (3)
C7—C8—H8	124.2	N5—C35—C36	100.2 (2)
S1—C8—H8	124.2	C45—C35—C36	113.2 (2)
N1—C9—N2	122.6 (3)	N5—C35—H35	109.8
N1—C9—S1	117.3 (2)	C45—C35—H35	109.8
N2—C9—S1	120.1 (2)	C36—C35—H35	109.8
N2—C10—C20	111.9 (2)	C37—C36—C35	104.0 (2)
N2—C10—C11	100.6 (2)	C37—C36—H36A	111.0
C20—C10—C11	115.1 (3)	C35—C36—H36A	111.0
N2—C10—H10	109.6	C37—C36—H36B	111.0
C20—C10—H10	109.6	C35—C36—H36B	111.0
C11—C10—H10	109.6	H36A—C36—H36B	109.0
C12—C11—C10	103.4 (3)	N6—C37—C38	122.2 (3)
C12—C11—H11A	111.1	N6—C37—C36	113.1 (3)
C10—C11—H11A	111.1	C38—C37—C36	124.7 (2)
C12—C11—H11B	111.1	C43—C38—C39	117.4 (3)
C10—C11—H11B	111.1	C43—C38—C37	121.0 (3)
H11A—C11—H11B	109.0	C39—C38—C37	121.6 (3)
N3—C12—C13	120.1 (3)	C40—C39—C38	120.5 (3)
N3—C12—C11	113.6 (3)	C40—C39—H39	119.7
C13—C12—C11	126.2 (3)	C38—C39—H39	119.7
C14—C13—C18	117.8 (3)	C41—C40—C39	121.4 (3)
C14—C13—C12	121.3 (3)	C41—C40—H40	119.3
C18—C13—C12	120.8 (3)	C39—C40—H40	119.3
C15—C14—C13	121.1 (3)	C42—C41—C40	118.1 (3)
C15—C14—H14	119.5	C42—C41—C44	120.7 (3)
C13—C14—H14	119.5	C40—C41—C44	121.2 (3)
C14—C15—C16	121.8 (3)	C41—C42—C43	121.1 (3)
C14—C15—H15	119.1	C41—C42—H42	119.4
C16—C15—H15	119.1	C43—C42—H42	119.4
C17—C16—C15	116.7 (3)	C38—C43—C42	121.5 (3)
C17—C16—C19	122.9 (3)	C38—C43—H43	119.2
C15—C16—C19	120.4 (3)	C42—C43—H43	119.2
C16—C17—C18	122.2 (3)	C41—C44—H44A	109.5
C16—C17—H17	118.9	C41—C44—H44B	109.5
C18—C17—H17	118.9	H44A—C44—H44B	109.5
C17—C18—C13	120.4 (3)	C41—C44—H44C	109.5
C17—C18—H18	119.8	H44A—C44—H44C	109.5
C13—C18—H18	119.8	H44B—C44—H44C	109.5
C16—C19—H19A	109.5	C46—C45—C50	118.2 (3)
C16—C19—H19B	109.5	C46—C45—C35	122.8 (3)
H19A—C19—H19B	109.5	C50—C45—C35	118.8 (3)
C16—C19—H19C	109.5	C45—C46—C47	121.5 (3)
H19A—C19—H19C	109.5	C45—C46—H46	119.3
H19B—C19—H19C	109.5	C47—C46—H46	119.3
C21—C20—C25	117.7 (3)	C48—C47—C46	117.9 (3)
C21—C20—C10	120.2 (3)	C48—C47—H47	121.1
C25—C20—C10	122.1 (3)	C46—C47—H47	121.1
C20—C21—C22	122.0 (3)	C49—C48—C47	123.0 (3)
C20—C21—H21	119.0	C49—C48—F2	118.7 (3)
C22—C21—H21	119.0	C47—C48—F2	118.3 (4)
C23—C22—C21	118.0 (3)	C48—C49—C50	118.6 (3)
C23—C22—H22	121.0	C48—C49—H49	120.7
C21—C22—H22	121.0	C50—C49—H49	120.7
C22—C23—C24	122.4 (3)	C49—C50—C45	120.8 (4)
C22—C23—F1	119.0 (4)	C49—C50—H50	119.6
C24—C23—F1	118.6 (4)	C45—C50—H50	119.6
			
C9—N2—N3—C12	−157.5 (2)	C10—C20—C25—C24	178.5 (3)
C10—N2—N3—C12	−7.5 (3)	C23—C24—C25—C20	1.6 (5)
C34—N5—N6—C37	−157.5 (3)	C31—C26—C27—C28	−0.2 (5)
C35—N5—N6—C37	−10.0 (3)	C32—C26—C27—C28	177.8 (3)
C6—C1—C2—C3	0.8 (5)	C26—C27—C28—C29	0.2 (5)
C7—C1—C2—C3	−177.7 (3)	C27—C28—C29—C30	−0.6 (6)
C1—C2—C3—C4	−2.0 (5)	C28—C29—C30—C31	1.0 (5)
C2—C3—C4—C5	1.8 (6)	C29—C30—C31—C26	−1.0 (5)
C3—C4—C5—C6	−0.5 (5)	C27—C26—C31—C30	0.5 (5)
C4—C5—C6—C1	−0.7 (5)	C32—C26—C31—C30	−177.4 (3)
C2—C1—C6—C5	0.5 (4)	C34—N4—C32—C33	1.2 (4)
C7—C1—C6—C5	179.0 (3)	C34—N4—C32—C26	−180.0 (3)
C9—N1—C7—C8	0.6 (3)	C31—C26—C32—C33	157.8 (3)
C9—N1—C7—C1	−178.6 (2)	C27—C26—C32—C33	−20.1 (5)
C6—C1—C7—C8	171.7 (3)	C31—C26—C32—N4	−20.9 (4)
C2—C1—C7—C8	−9.8 (4)	C27—C26—C32—N4	161.2 (3)
C6—C1—C7—N1	−9.2 (4)	N4—C32—C33—S2	−0.4 (4)
C2—C1—C7—N1	169.3 (3)	C26—C32—C33—S2	−179.1 (3)
N1—C7—C8—S1	−0.3 (3)	C34—S2—C33—C32	−0.3 (3)
C1—C7—C8—S1	178.8 (2)	C32—N4—C34—N5	175.9 (3)
C9—S1—C8—C7	0.0 (2)	C32—N4—C34—S2	−1.5 (4)
C7—N1—C9—N2	178.3 (2)	N6—N5—C34—N4	171.7 (3)
C7—N1—C9—S1	−0.7 (3)	C35—N5—C34—N4	28.0 (5)
N3—N2—C9—N1	166.1 (3)	N6—N5—C34—S2	−10.9 (4)
C10—N2—C9—N1	19.3 (4)	C35—N5—C34—S2	−154.7 (2)
N3—N2—C9—S1	−15.0 (3)	C33—S2—C34—N4	1.1 (3)
C10—N2—C9—S1	−161.7 (2)	C33—S2—C34—N5	−176.4 (3)
C8—S1—C9—N1	0.4 (2)	C34—N5—C35—C45	−81.5 (4)
C8—S1—C9—N2	−178.6 (2)	N6—N5—C35—C45	133.9 (3)
C9—N2—C10—C20	−78.9 (3)	C34—N5—C35—C36	157.5 (3)
N3—N2—C10—C20	133.5 (2)	N6—N5—C35—C36	12.9 (3)
C9—N2—C10—C11	158.3 (3)	N5—C35—C36—C37	−10.7 (3)
N3—N2—C10—C11	10.7 (3)	C45—C35—C36—C37	−132.0 (3)
N2—C10—C11—C12	−9.4 (3)	N5—N6—C37—C38	−177.7 (3)
C20—C10—C11—C12	−129.9 (3)	N5—N6—C37—C36	1.9 (4)
N2—N3—C12—C13	−177.4 (2)	C35—C36—C37—N6	6.2 (4)
N2—N3—C12—C11	0.4 (3)	C35—C36—C37—C38	−174.2 (3)
C10—C11—C12—N3	6.2 (3)	N6—C37—C38—C43	−177.0 (3)
C10—C11—C12—C13	−176.1 (3)	C36—C37—C38—C43	3.4 (5)
N3—C12—C13—C14	−0.7 (4)	N6—C37—C38—C39	3.1 (5)
C11—C12—C13—C14	−178.2 (3)	C36—C37—C38—C39	−176.5 (3)
N3—C12—C13—C18	178.6 (3)	C43—C38—C39—C40	0.2 (5)
C11—C12—C13—C18	1.1 (4)	C37—C38—C39—C40	−179.9 (3)
C18—C13—C14—C15	−2.6 (4)	C38—C39—C40—C41	−0.3 (5)
C12—C13—C14—C15	176.7 (3)	C39—C40—C41—C42	−0.1 (5)
C13—C14—C15—C16	0.6 (4)	C39—C40—C41—C44	−178.4 (3)
C14—C15—C16—C17	1.5 (4)	C40—C41—C42—C43	0.6 (5)
C14—C15—C16—C19	−176.8 (3)	C44—C41—C42—C43	179.0 (3)
C15—C16—C17—C18	−1.7 (4)	C39—C38—C43—C42	0.4 (5)
C19—C16—C17—C18	176.6 (3)	C37—C38—C43—C42	−179.6 (3)
C16—C17—C18—C13	−0.3 (4)	C41—C42—C43—C38	−0.8 (5)
C14—C13—C18—C17	2.4 (4)	N5—C35—C45—C46	−32.9 (4)
C12—C13—C18—C17	−176.9 (3)	C36—C35—C45—C46	80.5 (3)
N2—C10—C20—C21	−58.9 (4)	N5—C35—C45—C50	152.4 (3)
C11—C10—C20—C21	55.1 (4)	C36—C35—C45—C50	−94.2 (3)
N2—C10—C20—C25	120.4 (3)	C50—C45—C46—C47	0.3 (4)
C11—C10—C20—C25	−125.6 (3)	C35—C45—C46—C47	−174.5 (3)
C25—C20—C21—C22	1.7 (5)	C45—C46—C47—C48	0.9 (5)
C10—C20—C21—C22	−179.1 (3)	C46—C47—C48—C49	−1.7 (5)
C20—C21—C22—C23	−0.5 (5)	C46—C47—C48—F2	179.4 (3)
C21—C22—C23—C24	−0.2 (6)	C47—C48—C49—C50	1.2 (5)
C21—C22—C23—F1	178.0 (3)	F2—C48—C49—C50	−179.9 (3)
C22—C23—C24—C25	−0.4 (6)	C48—C49—C50—C45	0.1 (5)
F1—C23—C24—C25	−178.6 (3)	C46—C45—C50—C49	−0.8 (4)
C21—C20—C25—C24	−2.2 (5)	C35—C45—C50—C49	174.2 (3)

### Hydrogen-bond geometry (Å, º)

Cg1, Cg2, Cg3 and Cg4 are the centroids of the C26–C31, C45–C50, S2,N4,C32–C34 and C1–C6 rings, respectively.

**Table:**

*D*—H···*A*	*D*—H	H···*A*	*D*···*A*	*D*—H···*A*
C15—H15···*Cg*1	0.93	2.75	3.537 (3)	143
C19—H19*A*···*Cg*2^i^	0.96	2.94	3.596 (4)	127
C35—H35···*Cg*3^ii^	0.98	2.94	3.843 (3)	153
C42—H42···*Cg*4^iii^	0.93	2.86	3.567 (3)	134

Symmetry codes: (i) *x*, −*y*, *z*+1/2; (ii) *x*−1, *y*, *z*; (iii) *x*, −*y*+1, *z*−1/2.
